# *C9orf72* poly(glycine-alanine) knock-in mice exhibit mild rotarod and proteomic changes consistent with amyotrophic lateral sclerosis/frontotemporal dementia

**DOI:** 10.1093/braincomms/fcag087

**Published:** 2026-03-17

**Authors:** Carmelo Milioto, Mireia Carcolé, Matteo Zanovello, Mhoriam Ahmed, Raja S Nirujogi, Daniel Biggs, Martha J Roberts, Kyra Schweers, Alexander J Cammack, Paolo M Marchi, Eszter Katona, Idoia Glaria, Almudena Santos, Anny Devoy, Pietro Fratta, Dario R Alessi, Ben Davies, Linda Greensmith, Elizabeth M C Fisher, Adrian M Isaacs

**Affiliations:** UK Dementia Research Institute at UCL, University College London, London WC1E 6BT, UK; Department of Neurodegenerative Disease, UCL Queen Square Institute of Neurology, London WC1N 3BG, UK; Dipartimento di Scienze Farmacologiche e Biomolecolari ‘Rodolfo Paoletti’, University of Milan, Milan20133, Italy; UK Dementia Research Institute at UCL, University College London, London WC1E 6BT, UK; Department of Neurodegenerative Disease, UCL Queen Square Institute of Neurology, London WC1N 3BG, UK; Department of Neuromuscular Diseases, UCL Queen Square Institute of Neurology, London WC1N 3BG, UK; Department of Neuromuscular Diseases, UCL Queen Square Institute of Neurology, London WC1N 3BG, UK; Aligning Science Across Parkinson’s (ASAP) Collaborative Research Network, Chevy Chase, MD 20815, USA; Medical Research Council (MRC) Protein Phosphorylation and Ubiquitylation Unit, School of Life Sciences, University of Dundee, Dundee DD1 5EH, UK; Wellcome Centre for Human Genetics, University of Oxford, Oxford OX3 7BN, UK; UK Dementia Research Institute at UCL, University College London, London WC1E 6BT, UK; Department of Neurodegenerative Disease, UCL Queen Square Institute of Neurology, London WC1N 3BG, UK; UK Dementia Research Institute at UCL, University College London, London WC1E 6BT, UK; Department of Neurodegenerative Disease, UCL Queen Square Institute of Neurology, London WC1N 3BG, UK; UK Dementia Research Institute at UCL, University College London, London WC1E 6BT, UK; Department of Neurodegenerative Disease, UCL Queen Square Institute of Neurology, London WC1N 3BG, UK; UK Dementia Research Institute at UCL, University College London, London WC1E 6BT, UK; Department of Neurodegenerative Disease, UCL Queen Square Institute of Neurology, London WC1N 3BG, UK; UK Dementia Research Institute at UCL, University College London, London WC1E 6BT, UK; Department of Neurodegenerative Disease, UCL Queen Square Institute of Neurology, London WC1N 3BG, UK; UK Dementia Research Institute at UCL, University College London, London WC1E 6BT, UK; Department of Neurodegenerative Disease, UCL Queen Square Institute of Neurology, London WC1N 3BG, UK; Research Support Service, Institute of Agrobiotechnology, CSIC-Government of Navarra, Mutilva31192, Spain; UK Dementia Research Institute at UCL, University College London, London WC1E 6BT, UK; Department of Neurodegenerative Disease, UCL Queen Square Institute of Neurology, London WC1N 3BG, UK; Department of Neurodegenerative Disease, UCL Queen Square Institute of Neurology, London WC1N 3BG, UK; Department of Neuromuscular Diseases, UCL Queen Square Institute of Neurology, London WC1N 3BG, UK; UCL Queen Square Motor Neuron Disease Centre, UCL Queen Square Institute of Neurology, University College London, London WC1N 3BG, UK; Francis Crick Institute, London NW1 1AT, UK; Aligning Science Across Parkinson’s (ASAP) Collaborative Research Network, Chevy Chase, MD 20815, USA; Medical Research Council (MRC) Protein Phosphorylation and Ubiquitylation Unit, School of Life Sciences, University of Dundee, Dundee DD1 5EH, UK; Wellcome Centre for Human Genetics, University of Oxford, Oxford OX3 7BN, UK; Francis Crick Institute, London NW1 1AT, UK; Department of Neuromuscular Diseases, UCL Queen Square Institute of Neurology, London WC1N 3BG, UK; UCL Queen Square Motor Neuron Disease Centre, UCL Queen Square Institute of Neurology, University College London, London WC1N 3BG, UK; Department of Neuromuscular Diseases, UCL Queen Square Institute of Neurology, London WC1N 3BG, UK; UCL Queen Square Motor Neuron Disease Centre, UCL Queen Square Institute of Neurology, University College London, London WC1N 3BG, UK; UK Dementia Research Institute at UCL, University College London, London WC1E 6BT, UK; Department of Neurodegenerative Disease, UCL Queen Square Institute of Neurology, London WC1N 3BG, UK; UCL Queen Square Motor Neuron Disease Centre, UCL Queen Square Institute of Neurology, University College London, London WC1N 3BG, UK

**Keywords:** C9orf72, frontotemporal dementia FTD, amyotrophic lateral sclerosis ALS, knock-in mouse model, GA dipeptide repeat

## Abstract

A GGGGCC repeat expansion in *C9orf72* is the most common genetic cause of amyotrophic lateral sclerosis (ALS) and frontotemporal dementia (FTD). The repeat expansion is translated into five different dipeptide repeat proteins: poly(glycine-alanine) (polyGA), poly(glycine-proline) (polyGP), poly(glycine-arginine) (polyGR), poly(alanine-proline) (polyAP) and poly(proline-arginine) (polyPR). To investigate the effect of polyGA, which is the most abundant dipeptide repeat protein in patient brains, we used clustered regularly interspaced short palindromic repeats (CRISPR) and CRISPR associated nuclease 9 (Cas9) to insert 400 codon-optimized polyGA repeats immediately downstream of the mouse *C9orf72* start codon. This generated (GA)400 knock-in mice driven by the endogenous mouse *C9orf72* promoter, coupled with heterozygous *C9orf72* reduction. PolyGA remains soluble up to 18 months of age and (GA)400 mice develop subtle dysfunction characterized by impaired rotarod performance, without overt neuropathological alterations. Quantitative proteomics revealed polyGA expression caused protein alterations in the spinal cord, including changes in previously identified polyGA interactors. Our findings show that (GA)400 mice are a complementary *in vivo* model to better understand *C9orf72* ALS/FTD pathology and determine the specific role of individual DPRs in disease.

## Teaser

Milioto *et al.* develop C9orf72 poly(glycine-alanine) (polyGA) knock-in mice to model frontotemporal dementia and amyotrophic lateral sclerosis. Soluble polyGA is generated, which causes impaired rotarod ability and proteomic alterations that overlap with polyGA interactors but not other C9orf72 knock-in mouse model proteome changes, indicating a distinct response to polyGA.

## Graphical Abstract

**Graphical Abstract:**
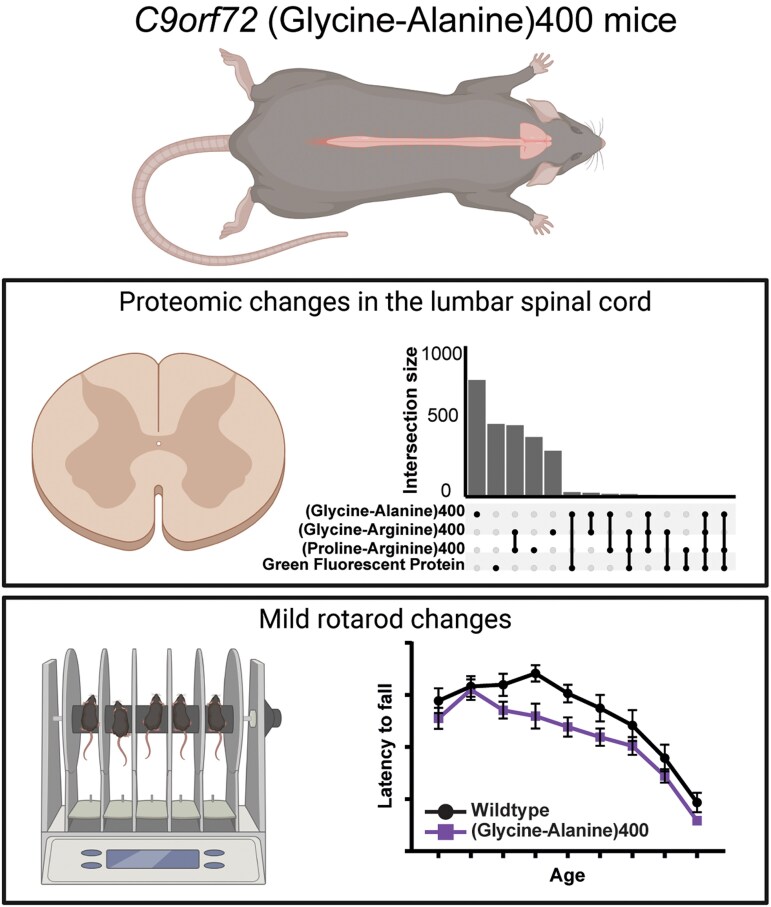

## Introduction

Amyotrophic lateral sclerosis (ALS) is the most frequent motor neuron disease in adults, characterized by progressive muscle weakness and atrophy leading to death typically from respiratory failure within a few years of diagnosis. Frontotemporal dementia (FTD) is the second most frequent young-onset dementia, characterized by progressive behavioural, language and cognitive impairment. Despite overtly different primary symptoms, ALS and FTD share clinical, neuropathological and genetic features. A hexanucleotide GGGGCC (G_4_C_2_) repeat expansion in the first intron of the *C9orf72* gene is the most common genetic cause of ALS and FTD, collectively termed C9ALS/FTD.^1-3^ Three mechanisms have been proposed to cause C9ALS/FTD pathology: (i) reduced transcription of *C9orf72*, (ii) the presence of sense and antisense repeat-containing RNA and (iii) expression of aberrant dipeptide repeat (DPR) proteins encoded in six frames by the hexanucleotide repeat.^4^

The presence of the hexanucleotide repeats gives rise to repeat-associated non-ATG initiated (RAN) translation, a non-canonical protein translation mechanism that does not require an ATG start codon.^5^ RAN translation occurs in every reading frame, encoding five different DPRs: poly(glycine-alanine) (polyGA), poly(glycine-proline) (polyGP), poly(glycine-arginine) (polyGR), poly(alanine-proline) (polyAP) and poly(proline-arginine) (polyPR), which all form neuronal cytoplasmic inclusions in C9ALS/FTD patient brains.^6,7^ Since the exact contribution of each DPR to disease pathogenesis is poorly understood, investigation of DPRs within the context of C9ALS/FTD is essential. We and others have demonstrated that DPR proteins are toxic *in vivo* and *in vitro*.^4,8^ A recent study of the interactome of DPRs identified interacting partner proteins involved in a variety of functions, including protein translation, signal transduction pathways, protein catabolic processes, amide metabolic processes and RNA-binding.^9^ Although the arginine-containing polyGR and polyPR proteins (R-DPRs) are the most toxic DPRs based on studies in several systems,^10-17^ considerable evidence also shows that DPRs not containing arginine induce toxicity. In particular, polyGA, the most abundant DPR species in patients, forms highly insoluble aggregates.^18-21^ Additionally, polyGA expression has been associated with TDP-43 phosphorylation and aggregation in cellular and mouse models, and this is a key feature in most human cases of ALS.^22-24^ Recently, polyGA overexpression in primary rat cortical neurons has been shown to promote loss of synaptic proteins and cause autophagy defects.^25^ Furthermore, analysis of cellular models and patient brain tissue revealed that polyGA aggregates sequester proteasome components, molecular chaperones and other proteins involved with protein folding/degradation pathways and nucleocytoplasmic transport, suggesting downstream defects in protein quality control and degradation.^26-29^

Several mouse models have been generated to elucidate how the presence of hexanucleotide repeats leads to neurodegeneration.^30,31^ Early studies showed that *C9orf72* homozygous knock-out mouse models, unlike heterozygous animals, develop severe autoimmunity and lymphatic defects, suggesting C9orf72 is involved in immune cell function.^32-39^ Mouse models of *C9orf72* hexanucleotide repeat expansions have been generated through AAV-mediated delivery,^40,41^ and bacterial artificial chromosome integration.^35,42-44^ Although most of these mouse models recapitulate aspects of C9ALS/FTD pathology, there is considerable variation between them, including the chromosomal site expressing the repeats, suggesting more refined models are needed.^45^ Additionally, several mouse models have been developed to better understand the role of specific DPRs. Studies of these mice show that *in vivo* expression of arginine-rich DPRs (R-DPRs) can drive toxicity.^13-17,46^ Similarly, studies in mice that over-express codon-optimized polyGA show that this can drive toxicity leading to neurodegeneration, motor and behavioural abnormalities and inflammation.^29,47^ Furthermore, expression of polyGA induces selective neuron loss, interferon responses and phosphoTDP-43 inclusions similar to those observed in C9ALS patients.^48^ Finally, in both cell and mouse models, antibodies targeting polyGA can reduce polyGA, polyGP and polyGR inclusions, improve behavioural deficits, decrease neuroinflammation and neurodegeneration, and increase survival.^49-52^

To date, multiple mechanisms have been implicated as common downstream molecular pathways in C9ALS/FTD, including autophagy, nucleocytoplasmic transport, pre-messenger RNA splicing, stress granule dynamics, DNA damage repair, mitochondrial dysfunction, nuclear pore alterations, impaired translation, lipid dyshomeostasis and synaptic dysfunction.^4,53-57^ However, despite considerable effort, the most relevant mechanism(s) by which the repeat expansion causes C9ALS/FTD remain unclear and no effective therapies have been developed to date. Thus, to understand the role(s) of individual DPRs *in vivo* and determine mechanisms associated with disease progression, we generated a series of *C9orf72* DPR knock-in mice. We recently described polyGR and polyPR knock-in mouse models that recapitulate many aspects of C9ALS/FTD pathology, such as cortical hyperexcitability and spinal motor neuron loss, and revealed a conserved neuroprotective extracellular matrix (ECM) signature in *C9orf72* ALS/FTD neurons.^58^ In that study we also generated control mice with eGFP knocked into *C9orf72* and they did not show any of these phenotypes, showing that the changes were due to expression of the DPRs. Here we describe polyGA knock-in mice, in which 400 polyGA repeats were inserted directly after the mouse *C9orf72* ATG start codon, resulting in a mouse model that expresses polyGA repeats from the *C9orf72* locus combined with heterozygous *C9orf72* reduction. We carried out phenotypic analyses of this mouse on a C57BL/6J background (the same background as our polyGR and polyPR knock-in animals) and found a mild phenotype, suggesting polyGA expression at a physiological level leads to subtle changes that are consistent with, but less pronounced, than seen in other *in vivo* and *in vitro* C9ALS/FTD experimental models.

## Materials and methods

### Assembly of targeting constructs

Assembly of targeting constructs was performed as previously described.^58^ Briefly, 100 codon-optimized polyGA repeats were synthesized (Thermo Fisher Scientific) and inserted into a pMC cloning vector containing 600 bp of the 5′ homology arm, a double haemagglutinin (HA)-tag, a V5 epitope tag, a simian virus 40 (SV40) polyA tail and 250 bp of 3′ homology arm. 400 polyGA repeats were then generated using recursive directional ligation and a selection cassette added followed by insertion into a targeting vector containing 2.7 kb 5′ and 3.2 kb 3′ homology arms as fully described previously when generating our (GR)400 and (PR)400 knock-in mice, which used exactly the same cloning strategy.^58^

### Animals

All procedures involving mice were conducted in accordance with the Animal (Scientific procedures) Act 1986, were performed at University College London under an approved UK Home Office project licence reviewed by the UCL Institute of Prion Diseases Animal Welfare and Ethical Review Body and were reported according to the ARRIVE2 guidelines, no *a priori* inclusion or exclusion criteria were set and no data were excluded. Mice were maintained in a 12 h light/dark cycle at a temperature of 20–24°C and relative humidity of 45–55% with food and water supplied *ad libitum*. The knock-in mice generated are available from the European Mutant Mouse Archive, EMMA ID EM:14600, strain name B6J.B6N-C9orf72^em1.1(GA400)Aisa^/H. Generation of the (GA)400 mouse strain was performed using exactly the same CRISPR assisted gene targeting strategy in JM8F6 embryonic stem (ES) cells that we used previously to generate our (GR)400 and (PR)400 knock-in mice.^58^ Germline-transmitting founders were obtained and backcrossed to wildtype C57BL/6J mice to maintain hemizygous lines for a minimum of five generations. Mouse genotype was determined by PCR for knock-in sequence with the following set of primers (forward 5′-TAAGCACAGCAGTCATTGGA-3′ and reverse 5′- AAGCGTAATCTGGAACATCG-3′). Repeat length was determined by PCR with the following set of primers (forward 5′-CCCATACGATGTTCCAGATTACGCTTACCC-3′ and reverse 5′-GCAATAAACAATTAGGTGCTATCCAGGCCCAG-3′). If not specified, males were used for experiments. Phenotyping was performed on both male and female mice.

### *C9orf72* 149R/2r AAV administration

To generate the 149 repeat (149R) model, C57BL6/J mice were injected at postnatal Day 0 (P0, within 24 h of birth) with AAV9 vectors encoding either 149 or 2 pure G4C2 repeats via intracerebroventricular injection into both hemispheres, as previously described.^41,59^ In brief, AAVs were first diluted in sterile PBS such that each animal received 6E10 vg in 4 µl total volume (3E10 vg in 2 µl per hemisphere). Then P0 pups were anaesthetized with isoflurane and AAVs were administered by manually injecting the diluted AAV solution with a calibrated Hamilton 10 µl syringe at a location approximately 2/5 of the distance from lambda to the eye and at a depth of approximately 2 mm. Post injection, pups were placed on a heat pad until fully recovered and then returned to the dam. Experimenters were blinded to AAV type before injection. Animals receiving 149R or 2R viruses were subsequently raised until either 1 or 6 months of age, at which point they were anaesthetized with isoflurane and perfused with ice-cold PBS. Brains were immediately removed and dissected and either snap-frozen on dry ice (for molecular biology) or drop-fixed in 4% PFA in PBS (for IHC). Both male and female animals were used in Mendelian ratios.

### Biochemical analysis

Brains and spinal cords were homogenized in lysis buffer (RIPA buffer (Pierce), 2% sodium dodecyl buffer (SDS), protease (Roche) and phosphatase (Thermo Fisher Scientific) inhibitors for total protein, and RIPA buffer with protease and phosphatase inhibitors for RIPA/urea fractionation). Lysates were sonicated and microcentrifuged for 20 min at 13 000 × g at room temperature and the supernatant was collected. For the RIPA/urea fractionation, the supernatant was ultracentrifuged for 30 min at 100 000 g at 4°C and the RIPA soluble fraction was collected. The pellets were homogenized in 8 M urea and ultracentrifuged again, after which the urea fraction was obtained. Proteins were separated on NuPAGE™ 4% to 12% bis-tris gels (Invitrogen) and transferred to nitrocellulose membranes (Bio-Rad Laboratories). Membranes were blocked in 5% milk in PBS-T (PBS, 0.1% Tween-20) for 1 h at room temperature. The membranes were incubated overnight at 4 °C with the following primary antibodies: C9orf72 (GTX634482, GeneTex, 1:1000), COL6 (ab182744, Abcam; 1:1000), Calnexin (sc-6465, Santa Cruz Biotechnology; 1:1000 dilution), GAPDH (14C10, #2118, Cell Signaling). After three washes in PBS-T, membranes were incubated with secondary HRP-conjugated antibodies for 1 h at room temperature. After three washes in PBS-T, signals were visualized by chemiluminescence (Amersham imager 680) and quantifications performed using ImageJ software. Uncropped blots are shown in the Supplementary material. Meso Scale Discovery (MSD) immunoassays were performed as previously described,^60-62^ using unlabelled anti-poly(GA) antibody (Sigma-Aldrich, #MABN889) as capture, and biotinylated anti-poly(GA) (GA5F2, kindly gifted by Prof Dieter Edbauer) as detector.

### Quantitative reverse transcription PCR

Tissues were dissected and flash frozen. Total RNA was extracted with an miRNeasy Micro Kit (Qiagen) and reverse-transcribed using SuperScript IV Reverse Transcriptase (Invitrogen) with random hexamers and Oligo(dT)_20_ primers. Gene expression was determined by quantitative real-time PCR (qPCR) using a LightCycler® and SYBR green (Roche). Relative gene expression was determined using the ΔΔCT method. Primers for mouse *C9orf72* are: 5′-TGAGCTTCTACCTCCCACTT-3′ and 5′-CTCTGTGCCTTCCAAGACAAT-3′. Primers to amplify the knock-in sequence are: 5′-GCGGCGAGTGGCTATTG-3′ (primer located within mouse *C9orf72* gene at exon boundary 1–2) and 5′-GGGTAAGCGTAATCTGGAACATC-3′ (sequence within the HA-tag sequence). Primers for mouse *GAPDH* are: 5′-TAGACAAAATGGTGAAGGT-3′ and 5′-AGTTGAGGTCAATGAAGG-3′.

### Immunohistochemistry

Mice were perfused with prechilled phosphate buffered saline (PBS) and then 4% paraformaldehyde (PFA). Brains and spinal cords were dissected and postfixed in 4% PFA at 4°C for 2 h. After fixation, the tissue was washed with PBS, allowed to sink in 30% sucrose solution at 4°C, then stored in 0.02% sodium azide at 4°C until further processing. Brains and spinal cords were embedded in optimal cutting temperature compound (Tissue Tek, Sakura, Torrance, CA), and 10 μm sections were cut with a cryostat (CM1860 UV, Leica Microsystem). For immunofluorescence, cryosections were washed 3 times in PBS and blocked in 5% BSA, 1% normal goat serum, 0.2% Triton-X in PBS for 1 h at room temperature. Sections were then incubated with primary antibodies in blocking solution overnight at 4°C. After three washes with PBS, sections were incubated for 1 h at room temperature in blocking solution with secondary antibodies conjugated with Alexa 488, 546, 594 and 633 (Invitrogen). After three washes in PBS, sections were mounted with ProLong™ Gold Antifade Mountant with DAPI (Invitrogen). The primary antibodies used were: IBA1 (019-19741, FUJIFILM Wako Pure Chemical Corporation; 1:500 dilution), GFAP (AB5804, Abcam; 1:500 dilution), CD68 (MCA1957, Bio-Rad Antibodies; 1:200 dilution), COL6 (ab182744, Abcam; 1:200), TDP43 (C-ter) (12892-1-AP, Proteintech, 1:400), P62 (23214, Cell Signaling, 1:400), GA (MABN889, Sigma-Aldrich, 1:100). Images were taken using a Zeiss LSM 880 confocal microscope, ZEISS Axio Scan.Z1 slide scanner or a Leica Mica Microhub. Image analyses were performed using ImageJ or QuPath software. Volumetric analysis of COL6A1 was performed following 3D reconstruction of the confocal images using Imaris software.

### Locomotor, grip strength and body weight assessment

Rotarod and grip strength assessment were performed as previously described.^58^ Tests were performed monthly from 3 to 9 months of age, and in 12- to 18-month-old mice. A power calculation using GPower predicted that for an effect size of 10% deviation from the group mean, with a power of 0.85 and an alpha of 0.05, groups sizes of 28 were needed, so we tested 14 females and 14 males per group. Body weight was measured weekly from 3 months of age. Mice were randomized into different experimental groups and the operator was blind to genotype.

### Motor neuron counts

Spinal cord ventral horn motor neurons were counted from L3 to L5 as described previously.^58^

### *In vivo* isometric muscle tension physiology

Isometric muscle tension physiology was performed as previously described.^63,64^

### Proteomic analysis

Mouse lumbar spinal cords and cortices were prepared in SDS-Lysis buffer (2% (weight/vol) SDS in 100 mM TEABC supplemented with Roche protease mini and Phos-STOP cocktail tablets) and analysed with an Orbitrap Tribrid Lumos mass spectrometer in-line with an Ultimate 3000 RSLC nano-liquid chromatography system as previously described for our (GR)400 and (PR)400 knock-in mice proteomics analyses.^58^

Data analysis for mouse tissue: spinal cord raw MS data was searched with Fragpipe software suite (version: 19.1)^65^ against the Uniprot Mouse database (April 2023) appended with (GA)400 sequences for C9orf72 and a common contaminant list exists within Fragpipe. FDR was set at 1% for both protein and PSM level. The protein group output files were further processed using Perseus software suite (version: 1.6.15.0)^66^ for downstream statistical analysis. Two-sided Welch’s *t*-Test with 5% FDR multiple-correction was performed to identify differentially regulated proteins. The previously analysed GR(400), PR(400) and Wildtype 15-plex TMT proteomics data^58^ was reprocessed as described above to match the database version and Fragpipe software suite used in the current study. This strategy has enabled us to map the protein accessions accurately between the datasets. Although, the DEPs may vary with set fold-difference cut-offs between the datasets due to the nature of batch-effects as commonly observed with multiple TMT-datasets.^67,68^ Gene Ontology analysis was performed on differentially regulated proteins using enrichR software.^69^ Gene Ontology analysis was performed on differentially regulated proteins using the software R and the package enrichR^69,70^(https://ggplot2.tidyverse.org). We assessed the co-occurrence of significant genes from our proteomics analysis with those found to be co-immunoprecipitated with polyGA in two different works,^9,57^ based on *Drosophila melanogaster* and *Homo sapiens* lines. First, we found mouse orthologues using DIOPT,^71,72^ then we represented the co-occurring genes using the software R and the package ggplot2^70^ (https://ggplot2.tidyverse.org).

### Statistical analysis

All data are presented as mean ± standard error of the mean (SEM). Statistical differences of continuous data from two experimental groups were calculated using unpaired two-sample Student’s *t*-test. Comparisons of data from more than two groups were performed using a one-way-ANOVA followed by Bonferroni correction for multiple comparisons. When two independent variables were available, comparisons of data from more than two groups were performed using a two-way-ANOVA followed by Bonferroni correction for multiple comparisons. Data distribution was tested for normality using Kolmogorov-Smirnov test; when normality could not be tested, we assumed data distribution to be normal. Statistical significance threshold was set at *P* < 0.05, unless otherwise indicated. Power calculations were performed to determine sample sizes.

### Data collection

Data collection and analysis were performed blind to the conditions of the experiments.

## Results

### Generation of *C9orf72* polyGA knock-in mice

**Figure 1:**
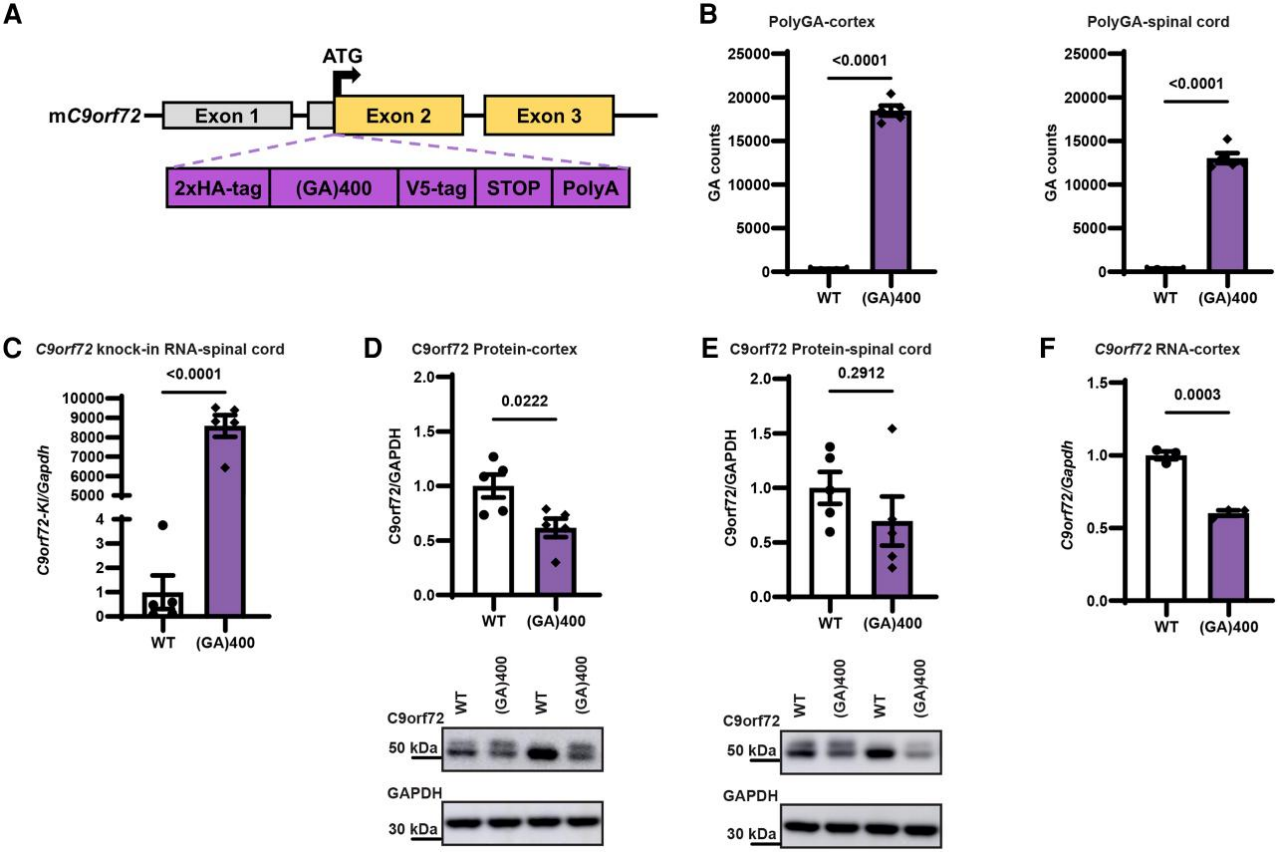
**Generation of *C9orf72* polyGA knock-in mice**. (**A**) Targeting strategy to generate (GA)400 mice with the knock-in sequence inserted into exon 2 of mouse *C9orf72* (*mC9orf72*) immediately after, and in frame with, the endogenous ATG. Schematic shows the genomic region and the knock-in targeting construct. Exons are shown boxed, untranslated regions of exons are coloured grey with translated regions in yellow. The targeting construct (in purple) contains the knock-in sequence composed of a double HA-tag, 400 codon-optimized GA repeats, a V5-tag, a stop codon, and a 120 bp polyA tail. (**B**) Quantification of polyGA proteins in cortex (left panel) and spinal cord (right panel) of wildtype (WT) and (GA)400 mice at 12 months of age by Meso Scale Discovery (MSD) immunoassay. Graph, mean ± SEM, *n* = 5 mice per genotype, two-sided unpaired two-sample Student’s *t*-test. Each individual data point represents a single mouse. (**C**) qPCR analysis of *C9orf72* knock-in transgene transcript levels normalized to glyceraldehyde-3-phosphate dehydrogenase *(Gapdh*) in spinal cord of WT and (GA)400 mice at 12 months of age. Primers in the HA-tag and upstream endogenous mouse sequence were used. Graph, mean ± SEM, *n* = 5 mice per genotype, two-sided unpaired two-sample Student’s *t*-test. Each individual data point represents a single mouse. (**D**) Western blotting analysis of C9orf72 protein levels in cortex of WT and (GA)400 mice at 12 months of age. GAPDH is shown as loading control. Graph, mean ± SEM, *n* = 5 mice per genotype, two-sided unpaired two-sample Student’s *t*-test. Each individual data point represents a single mouse. See Supplementary materials for uncropped blots. (**E**) Western blotting analysis of C9orf72 protein levels in spinal cord of WT and (GA)400 mice at 12 months of age. GAPDH is shown as loading control. Graph, mean ± SEM, *n* = 5 mice per genotype, two-sided unpaired two-sample Student’s *t*-test. Each individual data point represents a single mouse. See Supplementary materials for uncropped blots. (**F**) qPCR analysis of *C9orf72* transcript levels normalized to *Gapdh* in brain cortex of WT and (GA)400 at 9 months of age. Graph, mean ± SEM, *n* = 3 mice per genotype; two-sided unpaired two-sample Student’s *t*-test. Each individual data point represents a single mouse.

In order to generate polyGA knock-in mice we adopted the same approach we used recently to generate polyGR and polyPR knock-in mice, which we will collectively refer to as R-DPR mice.^58^ We first built patient-length, seamless, codon-optimized polyGA repeats by using recursive directional ligation^10,73^and flanked them with epitope tags (Fig. 1A). We then used CRISPR-Cas9 technology to insert the repeats directly after the endogenous mouse *C9orf72* ATG start codon in mouse ES cells (Supplementary Fig. 1A). We next used targeted locus amplification to identify targeted ES cells that had a single insertion site, correct targeting and no backbone co-integration (Supplementary Figs 1B–1D). Validated ES cell clones were then used to generate knock-in mice using standard procedures. Briefly, targeted ES cells (C57BL/6N) were injected into C57BL/6J blastocysts, and the resulting chimaeras were crossed to wildtype C57BL/6J mice for germline transmission, founders were backcrossed to C57BL/6J animals for a minimum of 5 generations before phenotypic analysis.

Heterozygous knock-in mice and littermate controls were aged and assessed, initially for polyGA expression. We detected polyGA in brain and spinal cord of (GA)400 animals at 12 months of age using an MSD immunoassay (Fig. 1B). As expected, at the same age, (GA)400 mice selectively expressed the knock-in sequence mRNA (Fig. 1C). As shown previously with our R-DPR knock-in mice,^58^ (GA)400 mice exhibited a significant ∼40% reduction in C9orf72 at both mRNA and protein levels in cortex and spinal cord compared with wildtype littermates (WT) mice (Fig. 1D-F). These results show that our (GA)400 mouse line expresses polyGA, alongside *C9orf72* reduction.

### (GA)400 mice develop mild rotarod dysfunction

**Figure 2:**
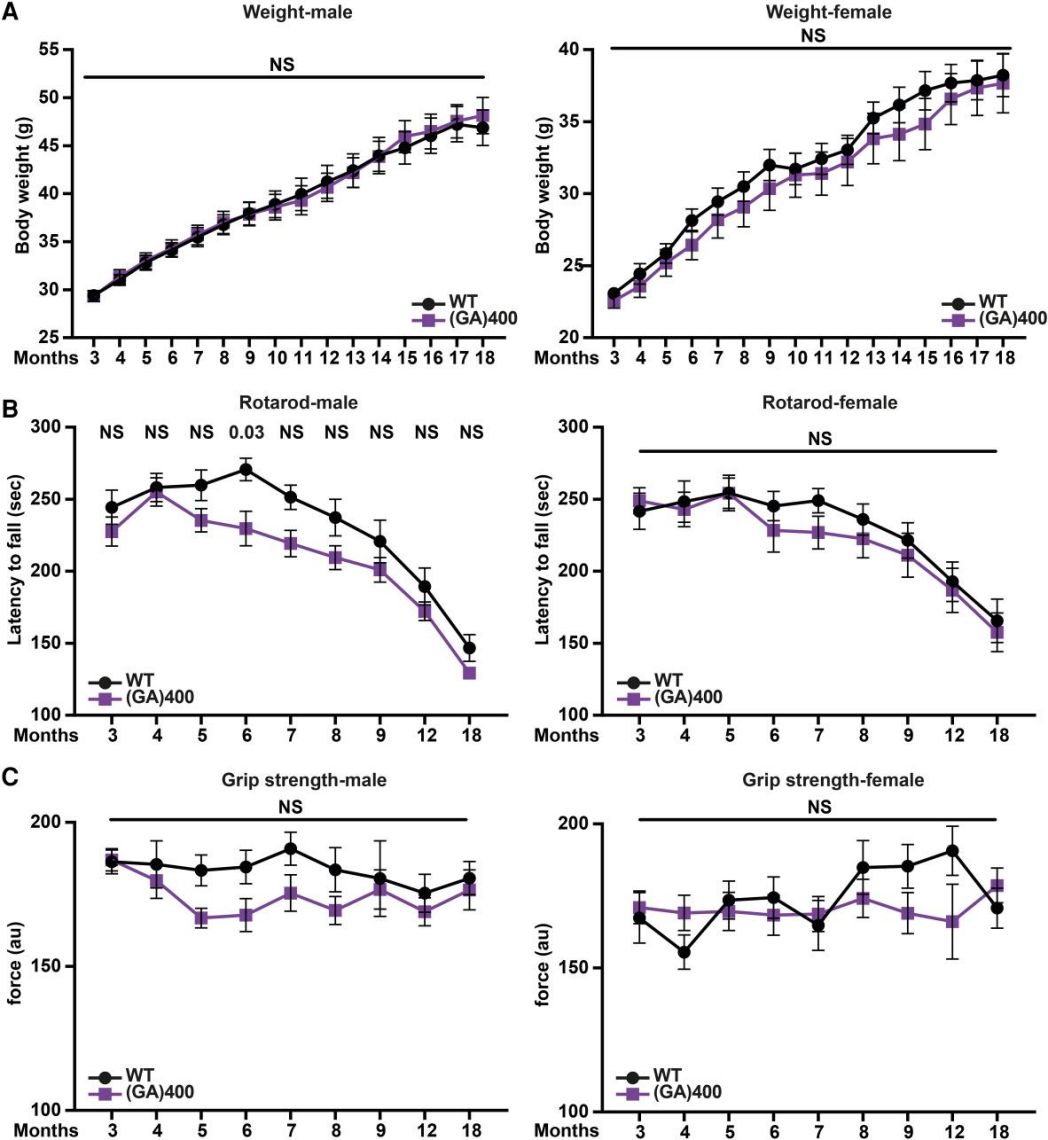
**(GA)400 mice develop mild motor dysfunction**. (**A**) Body weights of wildtype (WT) and (GA)400 male (left panel) and female (right panel) mice up to 18 months of age. Graph, mean ± SEM, *n* = 14 mice per genotype, two-way ANOVA, Bonferroni’s multiple comparison, NS denotes *P* > 0.05. (**B**) Accelerated rotarod analysis of motor coordination in WT and (GA)400 male (left panel) and female (right panel) mice up to 18 months of age. Graph, mean ± SEM, *n* = 14 mice per genotype, two-way ANOVA, Bonferroni’s multiple comparison, NS denotes *P* > 0.05. (**C**) Grip strength analysis of muscle force in WT and (GA)400 male (left panel) and female (right panel) mice up to 18 months of age (au—arbitrary units). Graph, mean ± SEM, *n* = 14 mice per genotype, two-way ANOVA, Bonferroni’s multiple comparison, NS denotes *P* > 0.05.

We next evaluated the motor function of (GA)400 mice. Monthly body weight measurements indicated that (GA)400 male and female animals did not differ from WT littermates up to 18 months of age (Fig. 2A). Next, we assessed motor coordination by conducting rotarod analysis from 3 to 18 months of age. Male (GA)400 mice performed significantly worse than their WT littermates in accelerated rotarod performance from 6 months of age, with female mice showing a similar but non-significant trend in the same direction (Fig. 2B). Additionally, we performed grip strength analysis and neither (GA)400 male nor female knock-in mice showed significant strength deficits up to 18 months of age, although (GA)400 males consistently performed less well than their littermate controls (Fig. 2C). Based on these results, we suggest that (GA)400 mice develop a milder dysfunction as they age, as compared with our previously characterized R-DPR knock-in mouse models.^58^

### PolyGA expression does not cause overt pathology up to 18 months of age

**Figure 3:**
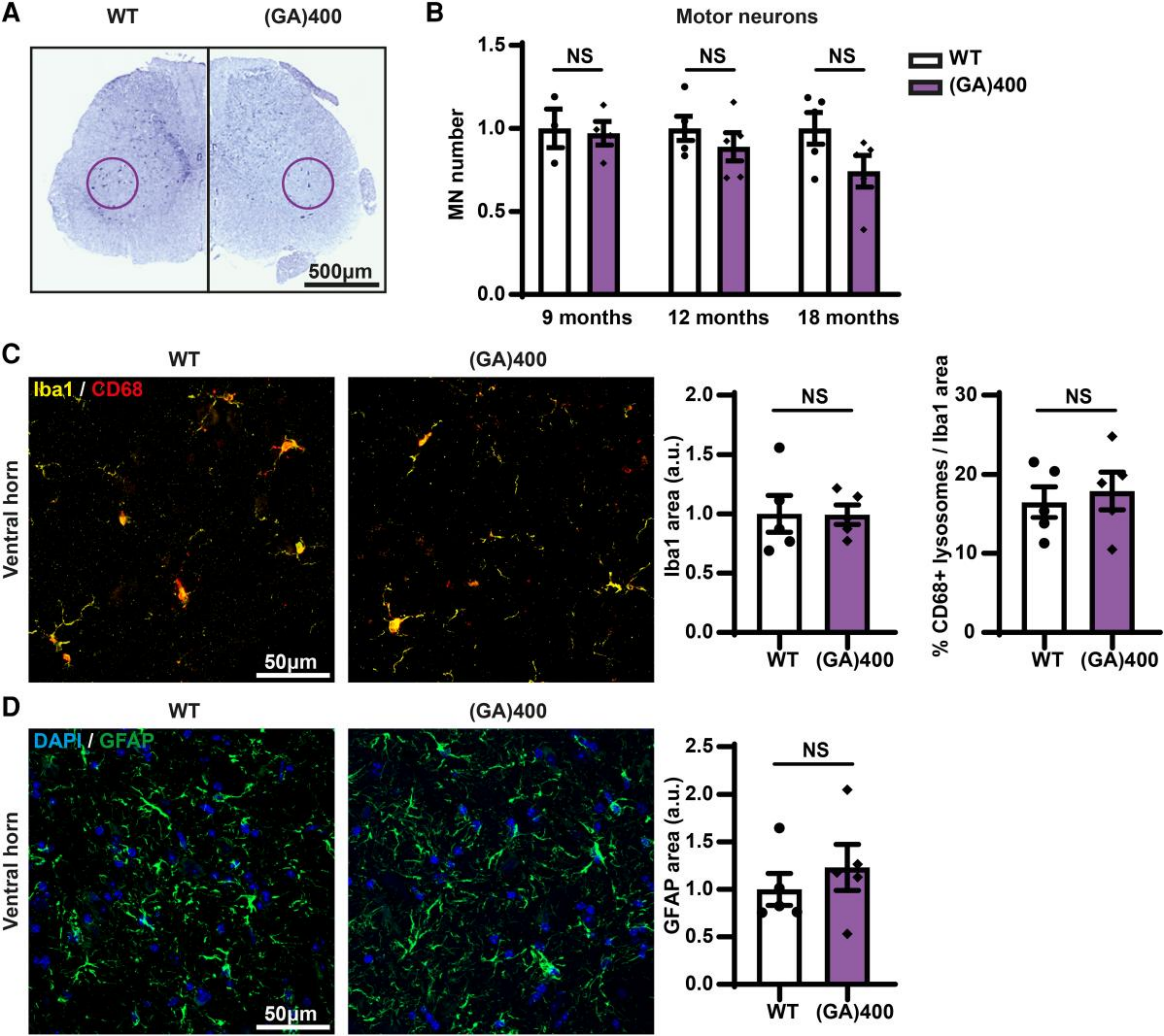
**Quantification of motor neurons and inflammation in (GA)400 mice**. (**A**) Panel shows representative image of Nissl staining of lumbar spinal cord in wildtype (WT) and (GA)400 mice at 18 months of age; the purple circle delineates the sciatic motor pool in which motor neurons were counted. (**B**) Quantification of Nissl-stained motor neurons in lumbar spinal cord region L3-L5 in WT and (GA)400 mice at 9, 12 and 18 months of age. Graph, mean ± SEM, *n* = 3–4 mice per genotype at 9 months, *n* = 5 mice per genotype at 12 months, *n* = 5 mice per genotype at 18 months, two-sided unpaired two-sample Student’s *t*-test, NS denotes *P* > 0.05. Each individual data point represents a single mouse. (**C**) Representative confocal images and quantification of immunofluorescence staining showing microglial density and colocalization between microglial markers ionized calcium-binding adaptor molecule 1 (Iba1) (yellow) and microglial lysosomal marker cluster of differentiation 68 (CD68) (red) in lumbar spinal cord ventral horn in WT and (GA)400 mice at 18 months of age. Graph, mean ± SEM, *n* = 5 mice per genotype, two-sided unpaired two-sample Student’s *t*-test, NS denotes *P* > 0.05. Each individual data point represents a single mouse. (**D**) Representative confocal images and quantification of immunofluorescence staining of astrocytic marker glial fibrillary acidic protein (GFAP) (green) in lumbar spinal cord ventral horn in WT and (GA)400 mice at 18 months of age. DAPI (4′,6-diamidino-2-phenylindole) (blue) stains nuclei. Graph, mean ± SEM, *n* = 5 mice per genotype, two-sided unpaired two-sample Student’s *t*-test, NS denotes *P* > 0.05. Each individual data point represents a single mouse.

We next investigated whether the subtle, but prolonged rotarod defect could be associated with motor neuron loss. We performed motor neuron counts at 9, 12 and 18 months of age in the ventral horn of the lumbar spinal cord as this is where the large alpha motor neurons reside that are primarily affected in ALS. We did not observe significant alteration in motor neuron numbers, but there was a non-significant trend towards lower motor neuron numbers as the mice aged compared with WT mice (Fig. 3A and B). In our R-DPR knock-in mice we had observed motor neuron loss at 12 months of age that was confirmed using electrophysiological motor unit number estimation (MUNE) analysis.^58^ To provide a direct comparison, we performed MUNE analysis in the hindlimbs of 12-month-old (GA)400 mice. Unlike our R-DPR knock-in mice, we did not observe a reduction in functional motor unit number in the extensor digitorum longus muscles of (GA)400 mice when compared with WT littermates (Supplementary Fig. 2A). Additionally, we investigated whether polyGA expression causes other ALS/FTD pathological hallmarks. We conducted immunostaining analysis in 18-month-old animals and did not observe microgliosis in the lumbar spinal cord (Fig. 3C) or cortex (Supplementary Fig. 2B) of (GA)400 mice. Similarly, we did not detect signs of astrogliosis in the lumbar spinal cord (Fig. 3D) or cortex (Supplementary Fig. 2C) of 18-month-old (GA)400 mice. Moreover, no signs of TDP-43 mis-localization were identified in cortex of 18-month-old mice (Supplementary Fig. 2D).

We also attempted to visualize polyGA using immunostaining but were unable to detect a signal using antibodies that are known to detect GA aggregate pathology.^18,74^ This indicates that the GA DPR in our mice is in a soluble form rather than the aggregates detected by currently available antibodies. To test this hypothesis we used the well characterized 5E9 anti-polyGA antibody that detects polyGA aggregates in patient brain.^18^ As a positive control we injected mice intracerebroventricularly at P0 with the well characterized (GGGGCC)149 AAV construct^41^ and performed polyGA and p62 immunostaining one month later. PolyGA and p62 aggregates were detected in the (GGGGCC)149 mice (Supplementary Fig. 3A-B**)**, showing that we are able to detect such aggregates if they are present. However, no polyGA or p62 aggregates were detected in (GA)400 mouse cortex or spinal cord at 18 months of age (Supplementary Fig. 3A-B**).** To further investigate the possibility that our inability to visualize polyGA is because it is not aggregated, we performed a two-step protein extraction in cortex and spinal cord lysates to generate soluble and insoluble protein fractions. We performed the same extraction protocol on 6-month-old (GGGGCC)149 cortex and measured polyGA using our MSD immunoassay. Consistent with our hypothesis, (GGGGCC)149 tissue was unique in having a significant proportion of polyGA in the insoluble fraction (Supplementary Fig. 3C**)**, while in (GA)400 mice polyGA remained in the soluble fraction. Based on these independent lines of evidence we suggest that polyGA in GA400 mice remains soluble precluding its visualization with antibodies that detect polyGA aggregates.

### PolyGA expression induces proteomic alterations in the spinal cord

**Figure 4:**
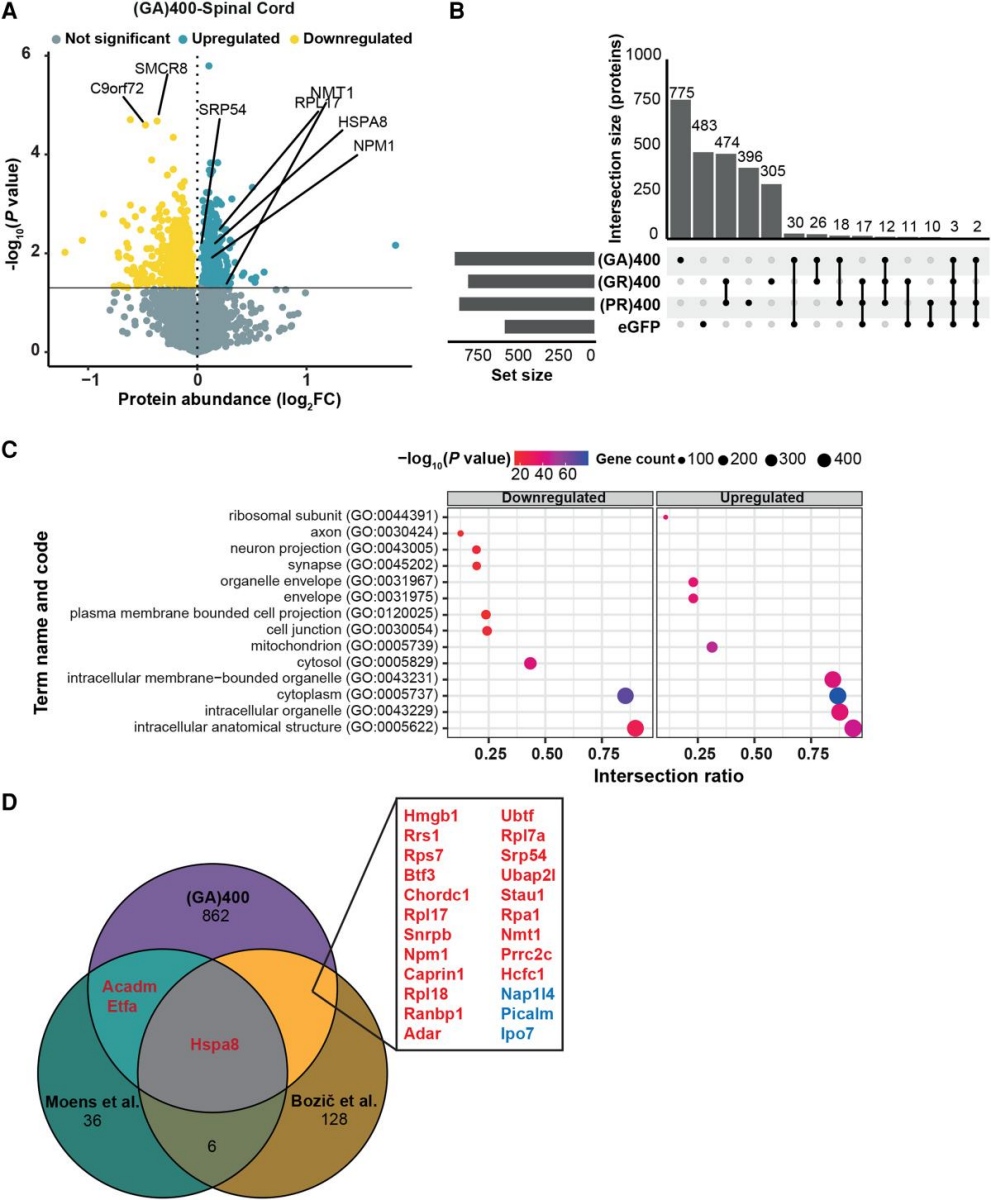
**PolyGA expression induces proteomic changes in the spinal cord**. (**A**) Protein expression volcano plots from the lumbar spinal cord of 12-month-old (GA)400. *n* = 5 mice per genotype, two-sided Welch’s *t*-Test with 5% false discovery rate (FDR) multiple-correction. (**B**) Upset plot showing the number and overlap of differentially expressed proteins (DEPs), compared with their respective littermate controls, in 12-month-old spinal cords of (GA)400 (GR)400, (PR)400 and *C9orf72* eGFP knock-in mice. DEPs were only considered shared if the direction of the change was the same. (**C**) Significantly enriched downregulated (left) and upregulated (right) Gene Ontology (GO) pathways in the lumbar spinal cord of 12-month-old (GA)400 mice. Proteomics performed on *n* = 5 mice per genotype, two-sided Welch’s *t*-Test with 5% FDR multiple-correction. The *x*-axis represents the intersection ratio between significant genes and the total number of genes for the specific GO category, while the size and colour of dots represent the total count and −log10(*P*-value) of significant genes for each category, respectively. (**D**) Venn diagram showing the overlap of GA(400) significant targets with the orthologues of the significant hits from Moens *et al*. ^57^ and Bozič *et al*.^9^ Overlapping targets are shown in red when upregulated in (GA)400 mice and in blue when downregulated.

We performed quantitative proteomics in the lumbar spinal cord of 12-month-old (GA)400 mice, which revealed modest changes at the protein level (Supplementary Table 1). Among these alterations were reduced levels of C9orf72 and its binding partner SMCR8 (Fig. 4A), which confirms the reduced C9orf72 levels we observed by western blotting (Fig. 1E).

We previously had observed an upregulation of ECM components in R-DPR knock-in mouse lumbar spinal cord at 12 months of age using the same quantitative proteomics approach.^58^ However, ECM proteins were not significantly increased in (GA)400 mice at 12 months of age (Supplementary Fig. 4A). As COL6A1 was the most increased ECM component in our R-DPR knock-in mice, we measured COL6A1 levels in the (GA)400 mice using immunoblotting. We did not detect alterations in COL6A1 protein level in cortex or spinal cord of 12-month-old (GA)400 mice (Supplementary Fig. 4B). Similarly, COL6A1 volume in spinal cord of 12-month-old (GA)400 mice was not altered (Supplementary Fig. 4C).

We next compared the (GA)400 proteomics data to all of our previously described *C9orf72* knock-in mice (Fig. 4B). This includes the R-DPR mouse models and *C9orf72* eGFP knock-in mice, where eGFP is knocked into the same location as the DPRs, and which serve as a control for the loss of one allele of *C9orf72*.^58^ All datasets were from 12-month-old lumbar spinal cords. We observed that the majority of differentially expressed proteins (DEPs) in (GA)400 mice were specific to (GA)400 (775 out of 866 DEPs were specific to (GA)400), with 30 shared with eGFP knock-in spinal cord, and 12 shared with (GR)400 and (PR)400 (Fig. 4B). In comparison, (GR)400 and (PR)400 mice shared the majority of their DEPs with each other (Fig. 4B), indicating a clear and common response to the R-DPRs that is distinct to that seen in (GA)400 mice.

Gene Ontology (GO) molecular processes term enrichment analysis of (GA)400 mice compared with wildtype mice revealed several significantly altered pathways in lumbar spinal cord, including downregulation of synaptic proteins (Fig. 4C), which were similarly downregulated in R-DPR knock-in spinal cord.^58^ Additionally, we compared our proteomic changes to recently published polyGA interactome studies in human cells^9^ and Drosophila^57^ (Fig. 4D). We found that HSPA8, a molecular chaperone previously associated with poly-GA aggregates and TDP-43 toxicity,^26,75^ was influenced by polyGA expression in all three models analysed. Notably, additional polyGA interactors, including NPM1, NMT1, RPL17 and SRP54 were significantly upregulated within our (GA)400 proteomic dataset (Fig. 4D). Comparison with the R-DPR and eGFP proteomic datasets showed that the polyGA interactor proteins are selectively altered in (GA)400 mice (Supplementary Table 2). This analysis suggests that physiological polyGA expression affects known polyGA interactor proteins *in vivo*. Thus, our polyGA knock-in mice exhibit proteomic changes observed in other polyGA models, but not in R-DPR mice, and may provide insight into early pathological changes selectively caused by polyGA.

## Discussion

Here, we describe a novel knock-in mouse to further investigate the role of DPR toxicity in C9ALS/FTD pathology. Using the same approach we previously adopted for R-DPR mice,^58^ we generated a mouse model that selectively expresses polyGA from the endogenous locus, with a reduced level of C9orf72, thus recapitulating two key features of *C9orf72* ALS/FTD. Briefly, we inserted a codon-optimized polyGA sequence immediately after and in frame with the mouse *C9orf72* ATG start codon. This strategy permits us to use the endogenous mouse *C9orf72* promoter and removes one *C9orf72* allele, thus reducing C9orf72 levels. Notably, we intentionally generated mouse models that produce each DPR in isolation. This means they do not model RAN translation or RNA toxicity, which will require other approaches. However, our reductionist approach allows a direct comparison of the effects of polyGA, polyGR and polyPR repeats in *in vivo* mouse models on a common genetic background.

(GA)400 mice exhibited a deficit in rotarod performance that was less prominent than in our (GR)400 and (PR)400 mice. Similarly, (GA)400 mice showed a non-significant trend towards motor neuron loss at 12 and 18 months of age, while (GR)400 and (PR)400 mice showed significant reductions in motor neurons at these time points. We interpret these results as showing that polyGA is not benign but is likely better tolerated and thus leads to milder effects than the R-DPRs, at least in the first 18 months of life in our mouse model. This supports evidence from several experimental models in which the arginine-rich polyGR and polyPR exerted stronger neurotoxic effects than polyGA.^3,10,30^

*C9orf72* repeat expansion can lead to FTD and we have not excluded the possibility of cognitive impairments in (GA)400 mice. It remains possible that the rotarod defect we observe is caused by cognitive rather than motor deficits, such as decreased motivation. Cognitive deficits remain of interest because Boyanova *et al.*^76^ have recently reported that our (GR)400 mouse model exhibits age-related deficits in short-term memory; the authors found no effect on total distance travelled by (GR)400 mice, confirming that their behavioural alterations are not due to effects on general locomotion. More research is needed to dissect these behavioural alterations, and they suggest that behavioural phenotypes should be investigated in (GA)400 mice in the future.

Based on our recent findings that expression of R-DPRs induces increased levels of specific ECM proteins in the spinal cord, which provide protection against neurodegeneration,^58^ we focused on spinal cord to identify potential mechanisms associated with polyGA expression. We performed proteomics analysis in the spinal cord at 12 months of age and analysed whether (GA)400 mice show the ECM signature we described in R-DPR mice and *C9orf72* iPSC-derived motor neurons. However, no significant change in ECM proteins was observed. Once more, these results are consistent with the milder effects observed in polyGA knock-in mice.

While the increased ECM signature was the most striking proteomic change in (GR)400 and (PR)400 mice, we also reported downregulated pathways with lower fold-changes. Due to the modest toxicity of polyGA, we did not expect proteomics analysis to reveal a strong similarity between (GA)400 and R-DPR mice. Surprisingly, we found several GO terms related to downregulated genes in the (GA)400 mouse that were shared with the R-DPR knock-in mouse models. Notably, these GO terms are related to neuronal and cellular homeostasis including synapse, neuron projection, plasma membrane bound cell projection, cell junction and axon, indicating neuronal/axonal or synaptic dysfunction. This observation suggests that each DPR causes neuronal dysfunction and raises the possibility that the individual DPRs might act synergistically in a C9ALS/FTD context to enhance toxicity.

Consistent with other knock-in models, the phenotypes were relatively modest across all our DPR knock-in mouse lines. This suggests that other factors are necessary in addition to DPR expression to drive the overt neuronal loss observed in patients. One possibility is an age-related decline in proteostasis that may allow higher levels of DPRs to accumulate over time, potentially closer to the levels utilized in overexpression studies that show overt toxicity and/or TDP-43 mislocalisation.^24,46,77^

Here, we show that (GA)400 mice, although sharing the same genomic background (C57BL/6J) with our (GR)400 and (PR)400 animals, develop more subtle changes than R-DPR mice. However, we should not rule out a role for polyGA in the context of ageing and proteostasis decline. In addition, a study that compared mice with conditional GFP-GA_175_ and GFP-PR_175_ inserted into the *Rosa26* safe harbour locus showed greater neurodegeneration and microglial activation in the polyGA line,^48^ indicating that in certain contexts polyGA confers clear toxicity. Indeed a mouse model overexpressing GFP-GA_50_ through AAV injection developed multiple phenotypes including astrogliosis, neuronal loss, ubiquitin-positive inclusions and behavioural deficits (such as hyperactivity and motor coordination defects).^29^ Conversely, a transgenic mouse model expressing (GA)_149_ with its endogenous C-terminal tail fused to cyan fluorescent protein is characterized by neuronal p62-positive poly(GA) inclusions and spinal microglial activation without neuronal loss or astrogliosis.^47^ These differences between different polyGA mice are most likely explained by differences in expression levels, with greater expression leading to neurodegeneration. Aggregation of polyGA has also been directly linked to its toxicity: GFP-GA_50_ caused neurodegeneration in mice, but inserting prolines into the GA_50_ sequence stopped it from aggregating and also rendered it unable to cause neurodegeneration.^29^ This could be relevant to recent clinical trials in which antisense oligonucleotides (ASOs) targeting mRNA transcripts harbouring the (G_4_C_2_) repeat expansion did not show clinical benefit.^78^ The ASOs successfully reduced soluble polyGA present in cerebrospinal fluid,^78^ but did not significantly reduce polyGA aggregates in patient brains.^79^ The mild symptoms we describe here, which are associated with soluble polyGA, indicate that removing aggregated GA may be essential for providing patient benefit. This has direct relevance for current therapeutic approaches focussing on polyGA removal by either passive or active immunization,^49,51,52^ whereby it now appears important for polyGA aggregates to be successfully removed.

Since we and others have previously performed polyGA interactome studies,^9,57^ we compared results obtained from these studies with our proteomics data. This comparison revealed that NPM1, NMT1, RPL17, SRP54 and HSPA8 were common across the datasets analysed. Interestingly, each of these proteins were upregulated in (GA)400 spinal cord. This could suggest a stabilizing interaction with polyGA, but further studies will be required to confirm this and whether there are functional consequences. Notably, this conservation of events across several models indicates a genuine phenomenon linked to the presence of polyGA.

Overall, our study shows that our novel (GA)400 mouse exhibits mild motor and proteomic changes that may model the earliest effects of polyGA in the brain and spinal cord. Together with our (GR)400 and (PR)400 mice, this series of DPR *C9orf72* knock-in mice provides a valuable tool to study the role of DPRs i*n vivo*.

## Supplementary Material

fcag087_Supplementary_Data

## Data Availability

The datasets generated and analysed during the present study are available from the corresponding authors upon request. Proteomics data including MS raw data and search output files have been deposited to the PRIDE ProteomeXchange Consortium via the PRIDE partner repository with the dataset identifier PXD047502.
