# Correction to: *C9orf72* poly(glycine-alanine) knock-in mice exhibit mild rotarod and proteomic changes consistent with amyotrophic lateral sclerosis/frontotemporal dementia

**DOI:** 10.1093/braincomms/fcag208

**Published:** 2026-06-05

This is a correction to: Carmelo Milioto, Mireia Carcolé, Matteo Zanovello, Mhoriam Ahmed, Raja S Nirujogi, Daniel Biggs, Martha J Roberts, Kyra Schweers, Alexander J Cammack, Paolo M Marchi, Eszter Katona, Idoia Glaria, Almudena Santos, Anny Devoy, Pietro Fratta, Dario R Alessi, Ben Davies, Linda Greensmith, Elizabeth M C Fisher, Adrian M Isaacs, *C9orf72* poly(glycine-alanine) knock-in mice exhibit mild rotarod and proteomic changes consistent with amyotrophic lateral sclerosis/frontotemporal dementia, *Brain Communications*, Volume 8, Issue 2, 2026, fcag087, https://doi.org/10.1093/braincomms/fcag087

In the originally published version of this manuscript, an incorrect grant number was listed in the Funding section.

Specifically, grant number “(MND020186)” should have been given as “(Isaacs/Apr20/876-791, Isaacs/Apr15/834-791)” in the following statement:

“This work was supported by the Motor Neurone Disease Association (MND020186) and the UK Dementia Research Institute (UK DRI) through UK DRI Ltd (UKDRI-1203), principally funded by the Medical Research Council; …” .

The statement has been corrected to read as follows:

“This work was supported by the Motor Neurone Disease Association (Isaacs/Apr20/876-791, Isaacs/Apr15/834-791) and the UK Dementia Research Institute (UK DRI) through UK DRI Ltd (UKDRI-1203), principally funded by the Medical Research Council; …”.

