# Supplementary material for: C9orf72 poly(glycine-alanine) knock-in mice exhibit mild rotarod and proteomic changes consistent with amyotrophic lateral sclerosis/frontotemporal dementia
Source: Brain Commun. 2026 Mar 17;8(2):fcag087. doi: 10.1093/braincomms/fcag087 (PMC13010074; doi:10.1093/braincomms/fcag087)
Supplement: fcag087_Supplementary_Data [file fcag087_supplementary_data.docx]

**Milioto*, Carcole* et al Supplementary Materials**


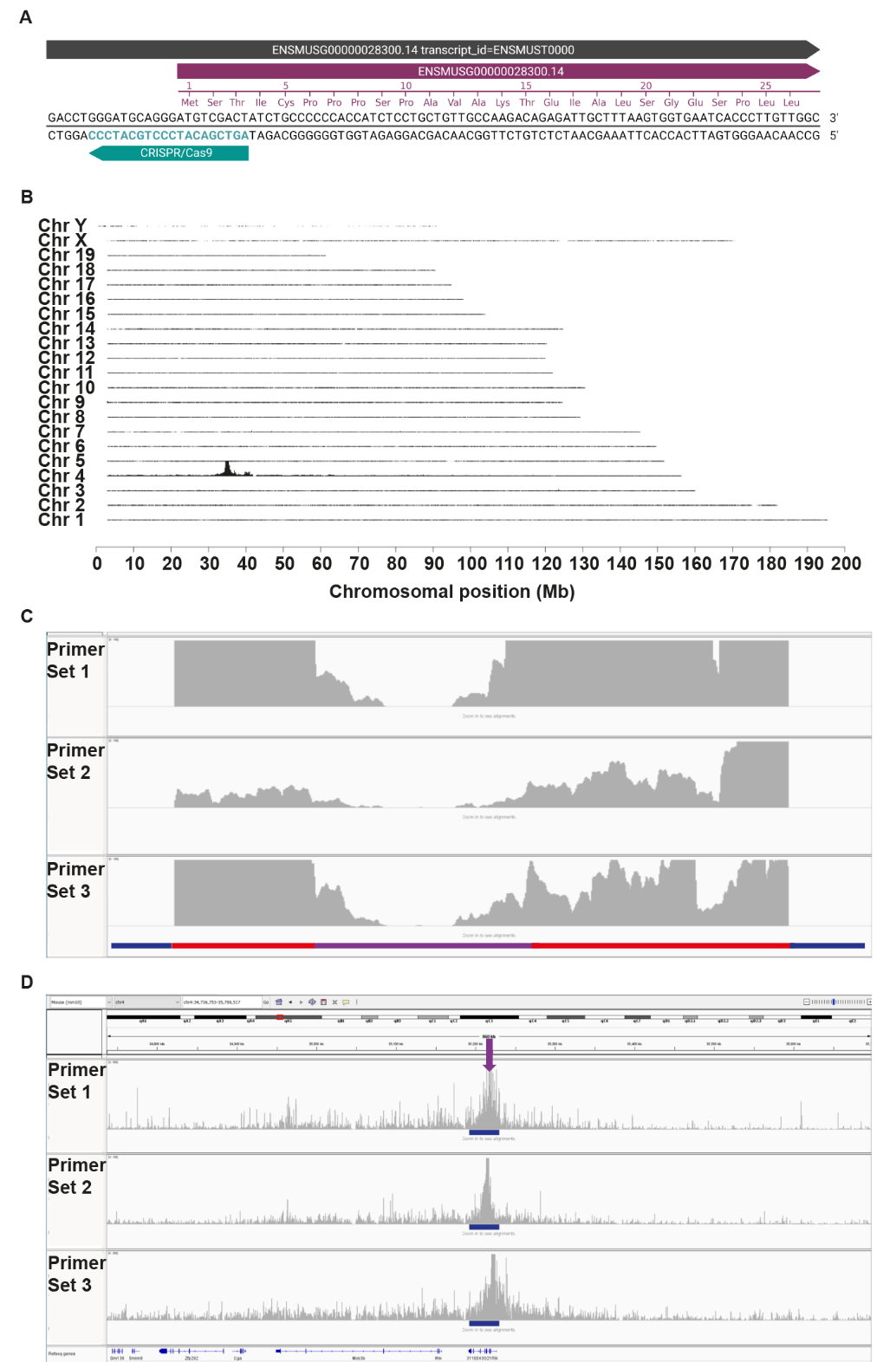


**Supplementary Figure 1.** ***C9orf72* knock-in strategy and confirmation. (A)** Design for Clustered Regularly Interspaced Short Palindromic Repeats (CRISPR) assisted *C9orf72* gene targeting. The sgRNA for CRISPR/Cas9 is indicated by the teal bar. **(B)** Mapping of targeted locus amplification reads across the mouse genome. The chromosomes (Chr) are indicated on the y-axis, the chromosomal position on the x-axis, Mb - megabases. **(C)** Targeted locus amplification sequence coverage across the knock-in sequence. The whole knock-in sequence has good coverage except for the blue underlined backbone sequences as expected from a correct targeting event as the backbone is not included. A coverage gap is present at the location of the 400 polyGA repeats and is underlined by the purple bar. The red bars mark the homology arms. Y-axis is limited to 100x. **(D)** Targeted locus amplification sequence coverage across the knock-in integration locus. The red bar marks the homology arms. The blue bar marks the Refseq Genes (3110043O21Rik). The purple arrow indicates the knock-in integration site. Y-axes are limited to 500x, 100x and 200x respectively.

**
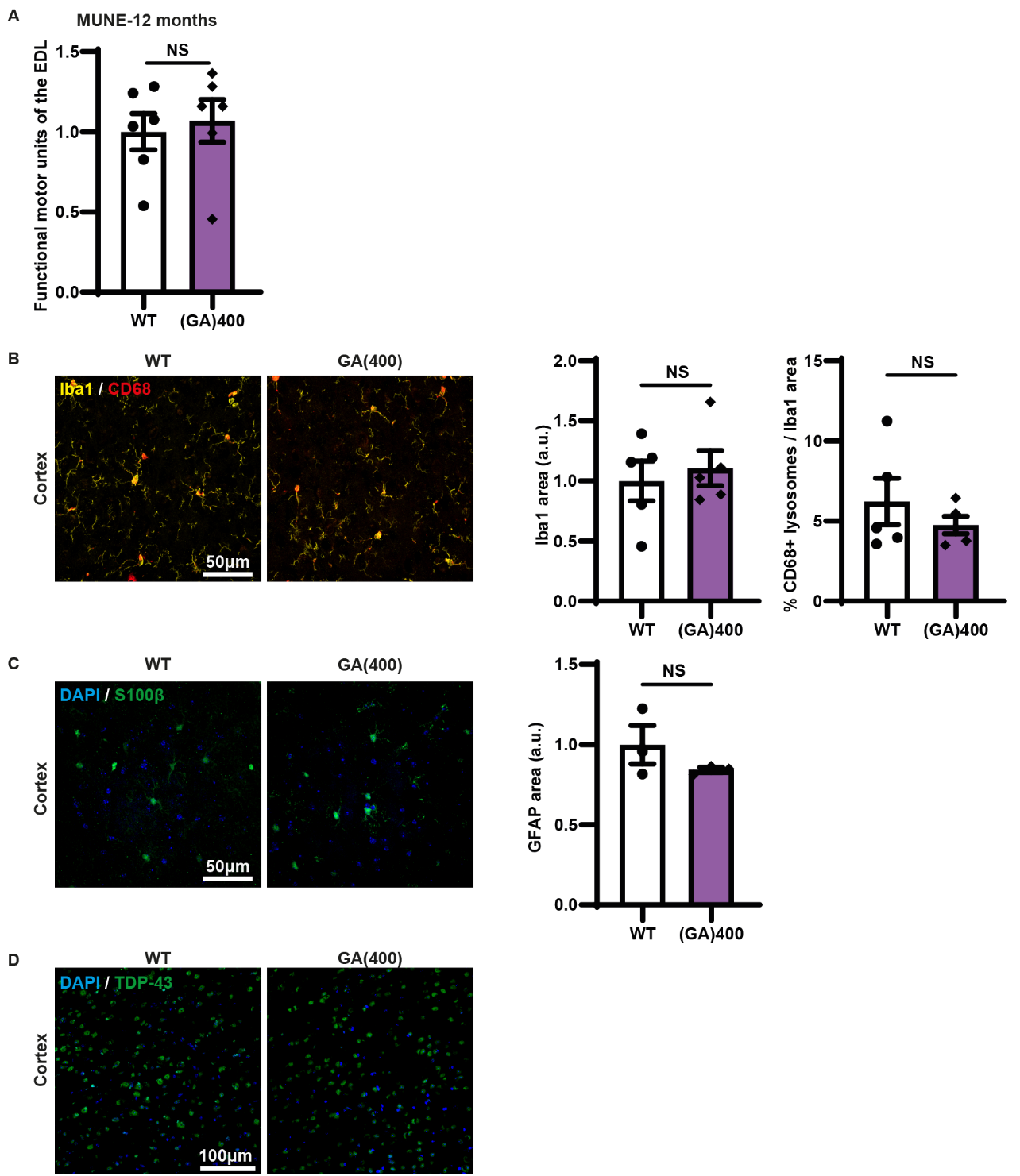
**

**Supplementary Figure 2.** **(GA)400 knock-in mice do not exhibit functional motor unit alteration at 12 months of age. (A)** Quantification of Motor Unit Number Estimation (MUNE) determined in Extensor Digitorum Longus (EDL) muscle in wildtype (WT) and (GA)400 mice at 12 months of age. Graph, mean ± SEM, n mice = 6 WT and 6 (GA)400, two-sided unpaired two-sample Student’s t-test, NS denotes *P* > 0.05. Each individual datapoint represents a single EDL muscle. **(B)** Representative confocal images and quantification of immunofluorescence staining showing microglial density and colocalization between microglial markers ionized calcium-binding adapter molecule 1 (Iba1) (yellow) and microglial lysosomal marker cluster of differentiation 68 (CD68) (red) in cortex in WT and (GA)400 mice at 18 months of age. Graph, mean ± SEM, n = 5 mice per genotype, two-sided unpaired two-sample Student’s t-test, NS denotes *P* > 0.05, a.u - arbitrary units. Each individual datapoint represents a single mouse. **(C)** Representative confocal images and quantification of immunofluorescence staining of astrocytic marker S100 calcium-binding protein B (S100β) (green) in cortex in wildtype (WT) and (GA)400 mice at 18 months of age. DAPI (4′,6-diamidino-2-phenylindole) (blue) stains nuclei. Graph, mean ± SEM, n = 3 mice per genotype, two-sided unpaired two-sample Student’s t-test, NS denotes *P* > 0.05. Each individual datapoint represents a single mouse. **(D)** Representative images of immunofluorescence staining showing Transactive Response DNA Binding Protein 43 kDa (TDP-43) (green) cellular localisation in brain cortex in wildtype (WT) and (GA)400 mice at 18 months of age. DAPI (blue) stains nuclei. n = 5 mice per genotype.

**
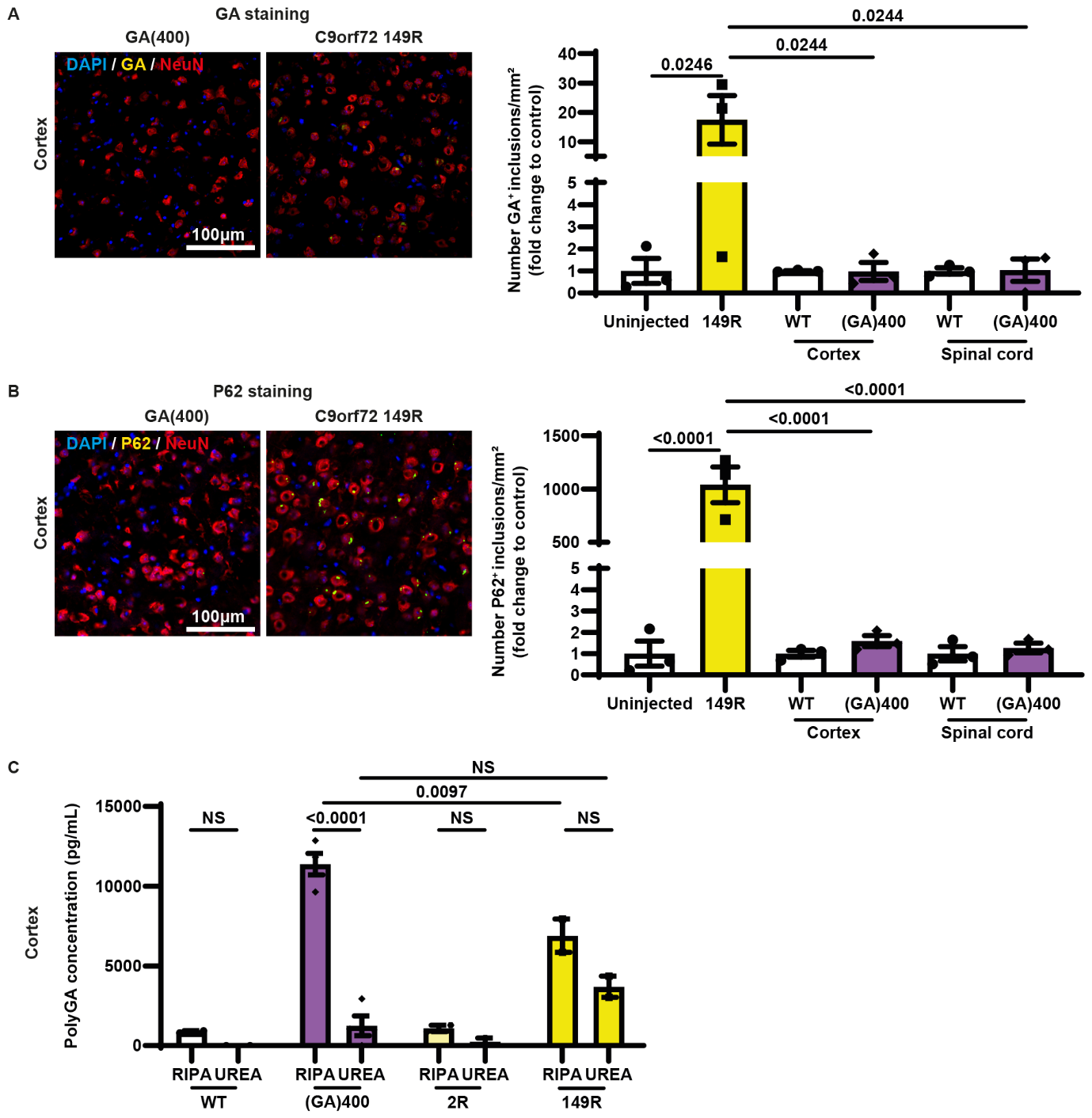
**

**Supplementary Figure 3.** **(GA)400 knock-in mice do not exhibit polyGA and p62 aggregates in cortex and spinal cord at 18 months of age. (A)** Representative confocal image and quantification of immunofluorescence staining of GA (yellow) in cortex of uninjected and (GGGGCC)149 AAV (149R) injected mice at 1 month of age, and cortex and spinal cord of wildtype (WT) and (GA)400 mice at 18 months of age. Neuronal Nuclei (NeuN) (red) stains neurons, DAPI (4′,6-diamidino-2-phenylindole) (blue) stains nuclei. Graph, mean ± SEM, n = 3 mice per genotype, one-way ANOVA, Bonferroni’s multiple comparison. Each individual datapoint represents a single mouse. **(B)** Representative confocal image and quantification of immunofluorescence staining of sequestosome 1 (p62) (yellow) in cortex of uninjected and 149R, cortex and spinal cord in WT and (GA)400 mice. NeuN (red) stains neurons, DAPI (blue) stains nuclei. Graph, mean ± SEM, n = 3 mice per genotype, one-way ANOVA, Bonferroni’s multiple comparison. Each individual datapoint represents a single mouse.

**(C)** Quantification of polyGA proteins in soluble (RIPA) and insoluble (urea) fractions in cortex of wildtype (WT) and (GA)400 mice at 18 months of age, with 2R and 149R at 6 months of age by Meso Scale Discovery (MSD) immunoassay. Graph, mean ± SEM, n = 2 for 2R, 149R, WT and n = 4 for (GA)400, two-way ANOVA, Sidak’s multiple comparison, NS denotes P > 0.05. Each individual datapoint represents a single mouse.

**
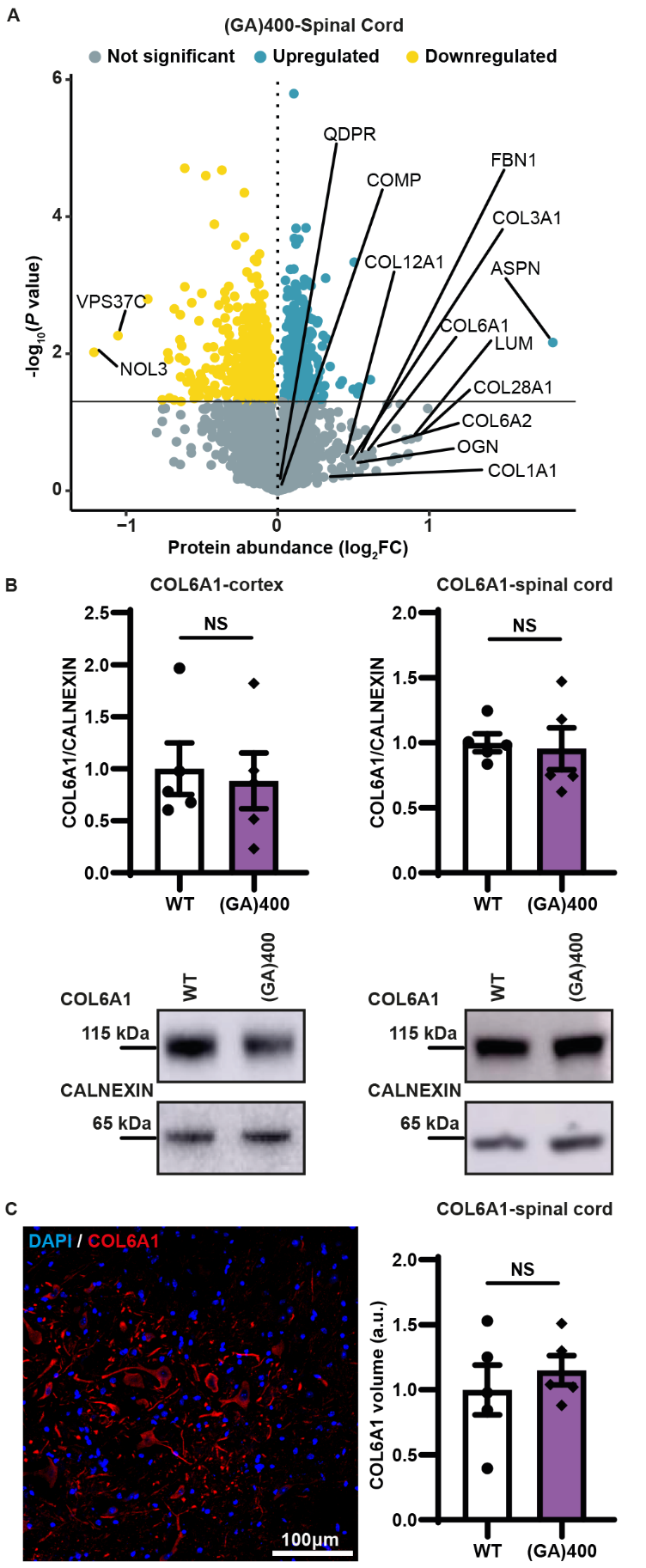
**

**Supplementary Figure 4. (GA)400 knock-in mouse spinal cord does not show increased extracellular matrix protein levels. (A)** Protein expression volcano plots from the lumbar spinal cord of 12-month-old (GA)400. n = 5 mice per genotype, two-sided Welch’s t-Test with 5% false discovery rate (FDR) multiple-correction. **(B)** Western blot of collagen alpha-1(VI) chain (COL6A1) in cortex (left panel) and lumbar spinal cord (right panel) of wildtype (WT) and (GA)400 mice at 12 months of age. Calnexin is shown as loading control. Graph, mean ± SEM, n = 5 mice per genotype, two-sided unpaired two-sample Student’s t-test, NS denotes *P* > 0.05. See supplementary material for uncropped blots. **(C)** Representative confocal image and volumetric quantification of immunofluorescence staining of COL6A1 (red) in lumbar spinal cord ventral horn in WT and (GA)400 mice at 12 months of age. DAPI (4′,6-diamidino-2-phenylindole) (blue) stains nuclei. Graph, mean ± SEM, n = 5 mice per genotype, two-sided unpaired two-sample Student’s t-test, NS denotes *P* > 0.05.

**Uncropped blots**


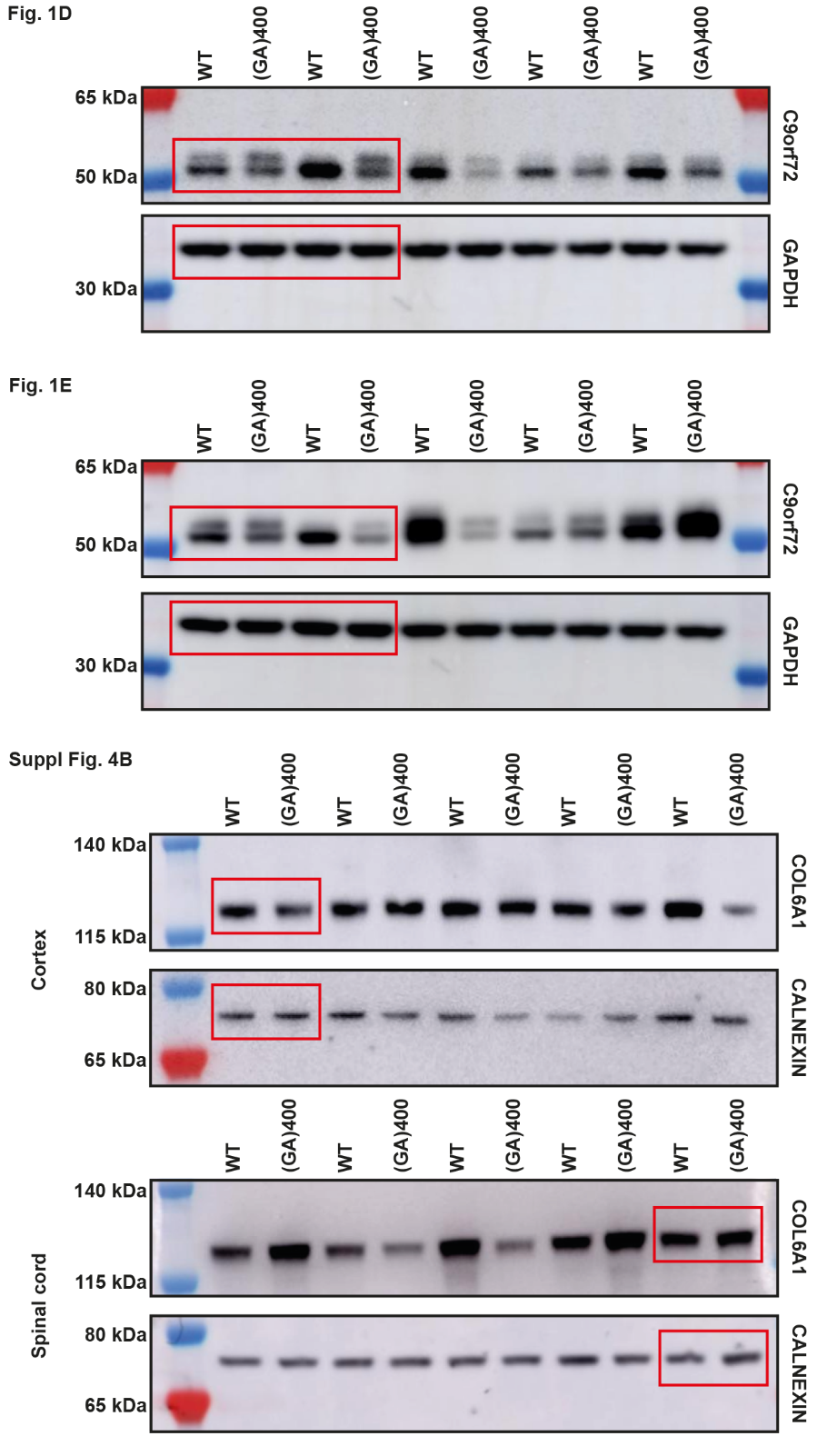

**Supplementary Table 1. Top 50 differentially expressed proteins in the lumbar spinal cord of 12-month-old (GA)400 mice.** Quantitative proteomics was performed on (GA)400 mice, shown as p-value and fold change vs littermate controls, (red highlight for significantly upregulated proteins, blue highlight for significantly downregulated proteins). N=5 mice per genotype. Also included for comparison are the p-values and fold change values for our previously published (GR)400, (PR)400 and eGFP *C9orf72* knock-in mouse 12-month-old lumbar spinal cord quantitative proteomics versus their respective littermate controls [1]. NA = not annotated in the dataset.

**Supplementary Table 2. Intersection of (GA)400 quantitative proteomics and previously published polyGA interactomes.** Differentially expressed proteins from quantitative proteomics in the lumbar spinal cord of 12-month-old (GA)400 mice versus littermate controls, which overlap with the orthologues of the significant hits from Moens et al., 2019 [2] and Bozič et al., 2022 [3] polyGA interactome studies (red highlight for significantly upregulated proteins, blue highlight for significantly downregulated proteins). Also included for comparison are the p values and fold change for our previously published (GR)400, (PR)400 and eGFP *C9orf72* knock-in mouse 12-month-old lumbar spinal cord quantitative proteomics versus their respective littermate controls [59]. NA = not annotated in the dataset.

**References**

1 Milioto C, Carcolé M, Giblin A, et al. PolyGR and polyPR knock-in mice reveal a conserved neuroprotective extracellular matrix signature in C9orf72 ALS/FTD neurons. *Nat Neurosci 2024*. Published online February 29, 2024:1-13. doi:10.1038/s41593-024-01589-4

2 Moens TG, Niccoli T, Wilson KM, et al. C9orf72 arginine-rich dipeptide proteins interact with ribosomal proteins in vivo to induce a toxic translational arrest that is rescued by eIF1A. *Acta Neuropathol*. 2019;137(3):487-500. doi:10.1007/S00401-018-1946-4

3 Bozič J, Motaln H, Janez AP, et al. Interactome screening of C9orf72 dipeptide repeats reveals VCP sequestration and functional impairment by polyGA. *Brain*. 2022;145(2):684-699. doi:10.1093/BRAIN/AWAB300
